# Durability Characteristics Analysis of Plastic Worm Wheel with Glass Fiber Reinforced Polyamide

**DOI:** 10.3390/ma6051873

**Published:** 2013-05-10

**Authors:** Gun-Hee Kim, Jeong-Won Lee, Tae-Il Seo

**Affiliations:** 1 Molds & Dies Technology R&D Group, Korea Institute of Industrial Technology, 7-47, Songdo-dong, Yeonsu-gu, Incheon 406-840, Korea; E-Mail: venkey@kitech.re.kr; 2 Division of Mechanical System Engineering, University of Incheon, 12-1, Songdo-dong, Yeonsu-gu, Incheon 406-772, Korea; E-Mail: tiseo@incheon.ac.kr

**Keywords:** glass fiber-reinforced plastic, plastic gear, durability test, injection molding

## Abstract

Plastic worm wheel is widely used in the vehicle manufacturing field because it is favorable for weight lightening, vibration and noise reduction, as well as corrosion resistance. However, it is very difficult for general plastics to secure the mechanical properties that are required for vehicle gears. If the plastic resin is reinforced by glass fiber in the fabrication process of plastic worm wheel, it is possible to achieve the mechanical properties of metallic material levels. In this study, the mechanical characteristic analysis of the glass-reinforced plastic worm wheel, according to the contents of glass fiber, is performed by analytic and experimental methods. In the case of the glass fiber-reinforced resin, the orientation and contents of glass fibers can influence the mechanical properties. For the characteristic prediction of plastic worm wheel, computer-aided engineering (CAE) analysis processes such as structural and injection molding analysis were executed with the polyamide resin reinforcement glass fiber (25 wt %, 50 wt %). The injection mold for fabricating the prototype plastic worm wheel was designed and made to reflect the CAE analysis results. Finally, the durability of prototype plastic worm wheel fabricated by the injection molding process was evaluated by the experimental method and the characteristics according to the glass fiber contents.

## 1. Introduction

MDPS (motor-driven power steering), a drive-steering system device that functions by means of a motor, is superior to the existing HPS (hydraulic power steering) in that it reduces the driver’s workload for operating the steering wheel. Thus, the percentage of automobiles with MDPS has been drastically increasing recently [1]. Unlike HPS, which assists steering by using the oil pressure generated by existing hydraulic motors, MDPS assists steering wheel operation with electric motor driving, which realizes the optimal steering power for each running speed range through the precise electric control of the motors and secures excellent stability of high-speed running through reaction and feedback to external forces.

**Figure 1:**
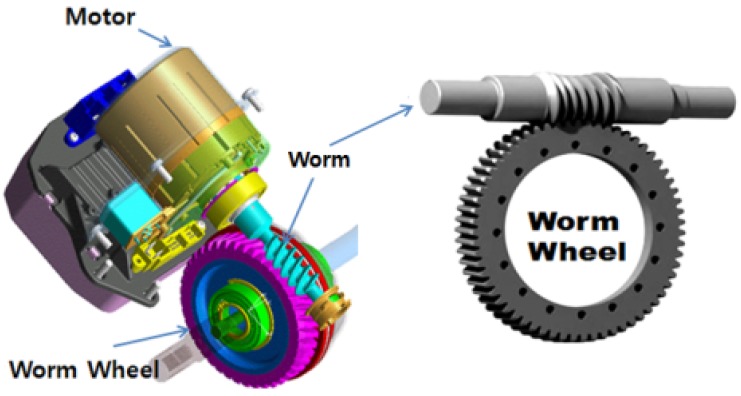
Worm and worm wheel system in vehicle steering reduction module of speed.

Basically, MDPS consists of an ECU (engine control unit) that assists the steering control unit, the torque sensor, and the reduction gear module that generates compensation torque. The reduction gear module (Figure 1) consists of a motor, a worm and worm wheel set, a backlash compensation apparatus, and so forth. The required specifications are the accuracy, durability, and hardness of the power transmission, as it is the core module part in direct relation to steering rotation. In particular, the worm and worm wheel parts that deliver power from the reduction gear module must secure the appropriate hardness, durability, and weight lightening, as well as noise and vibration reduction. Existing gear parts are mostly made of metals to secure hardness, which results in disadvantages such as high production cost due to the low productivity, poor corrosion resistance, vibration and noise generated, and so forth. Therefore, as the technology for plastic materials and injection molding has developed recently, interest has been aroused for the production of gear parts made of functional plastic materials [2,3,4,5,6]. Plastic gears can be suitable for mass production in terms of production and manufacturing, can be molded with other parts as one body, and have outstanding functional features in terms of vibration reduction, light weight, and corrosion resistance. Due to vehicle weight, however, this type of system has some disadvantage, as well, regarding hardness and durability against load for steering operation, and thus a lot of attention must be paid to securing hardness and durability in designing and manufacturing gears. For the supplement of plastic gear disadvantages, various researches have been performed. Specifically, Hoskins *et al.* [7] investigated how the generated sound frequency spectrum is influenced by the various polymeric gear materials and operating conditions. In this research, results also demonstrated the influence of increases of surface roughness, wear, and temperature on the respective sound power levels. Senthilvelan *et al.* [8] carried out the analysis on unreinforced Nylon 6/6, 20% short glass and 20% carbon fiber-reinforced Nylon 6/6 gear materials that indicates the reduction of the damping factor due to the incorporation of fibers. In the results of this study, it is indicated that the reinforced gears generate more gear mesh noise than unreinforced gears. In addition to another study by Senthilvelan [9], unreinforced and 20% short glass fiber-reinforced Nylon 6/6 spur gears were injection molded in the laboratory, and computer-aided simulations of gear manufacturing was carried out. Mao *et al.* [10] researched an extensive investigation of acetal and nylon gear friction and wear behavior. In this study, tests were performed using the acetal pinion with acetal gears, and nylon pinions with nylon gears, with further investigation carried out using dissimilar polymer gears. In the test results, it was found that the surface temperature was the dominant factor influencing the wear rate, and the initial relationship between the gear surface temperature was the dominant factor influencing the wear rate and an initial relationship between gear surface temperature and gear load capacity has been established and further developed.

This study aims to design, produce, and evaluate the performance of a plastic worm wheel that can be applied to an MDPS reduction gear module for compact and family sedan vehicles. Prior to the plastic worm wheel design, the worm to be combined with the worm wheel was designed, and so were the corresponding spur and the helical plastic worm wheels. Subsequently, the gear tooth profile and design plan were verified through structural computer-aided engineering (CAE) analysis and the injection mold design and molding process were examined through injection molding CAE analysis. The plastic gear was produced by injection molding with functional engineering plastic resin to which glass fiber reinforcement was added. Lastly, its durability and hardness were evaluated experimentally.

## 2. Design of Plastic Worm Wheel and Injection Mold

### 2.1. Design of Plastic Worm Wheel and Structure Analysis

**Figure 2:**
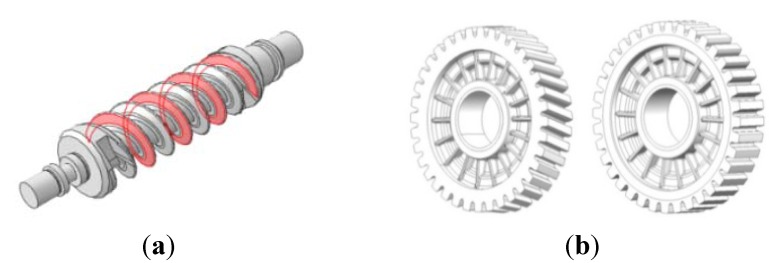
Design of worm and worm wheel: (**a**) worm; (**b**) worm wheel with helical and spur tooth form.

As for the existing gears adopted as automobile parts, only the tooth profile parts are molded with plastic resin, usually with a metal hub inserted. In contrast, this study designs a hub-less type of plastic worm wheel that removes the need for a metal hub, and most of the gear consists of plastic resin. This type of gear is advantageous in terms of its weight-lightening capacity and has no need for a separate heat treatment and finish-cutting process, which reduces the production cost. In addition, this prevents defects due to separation from the metal material, and enhances the accuracy of the tooth profile by skipping the finishing process, as the injection molding covers the complete process of gear tooth profiling. However, it is necessary to design a gear structure that minimizes potential contraction after molding and to secure structural strength. Table 1 shows the specifications and design values of the spur and helical worm wheels designed in this study. Figure 2 shows the plan for the worm wheel to be combined with the designed spur and the helical worm wheels.

**Table 1:** The hub-less worm wheel specification with spur and helical tooth form.

*Item*	*UNIT*	*Spur-type*	*Helical-type*
Pressure angle	degree	14.5	14.0
Addendum	mm	−0.36	−0.35
Dedendum	mm	2.64	2.65
Tooth thickness	mm	1.57	1.57
Normal module	mm	2.37	2.28
Helix angle	degree	0.0	16.58
Transverse pressure angle	degree	14.5	14.58
Tip diameter	mm	92.5	92.53
Reference diameter	mm	87.8	88.09
Root diameter	mm	81.74	82.02
Base diameter	mm	84.97	85.18
Normal tooth thickness	mm	5.4	5.12
Tip radius	mm	0.35	0.35
Base tangent length	mm	20.13	26.26
Number of teeth spanned	ea	3	4
Center distance	mm	52	52
Shaft angle	degree	73	90
Number of teeth	ea	36	36

**Figure 3:**
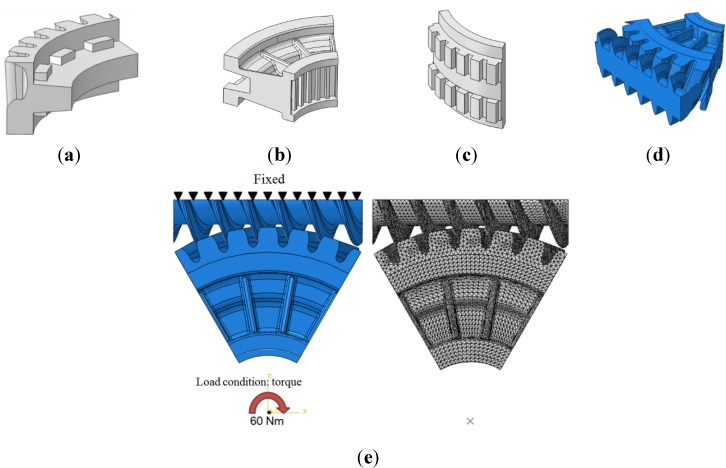
Assemble model for strain-stress CAE analysis. (**a**) tooth form part; (**b**) middle locking part; (**c**) end part; (**d**) tooth, middle locking, end parts and worm gear assembly; (**e**) boundary and load conditions and finite element model.

The worm wheel is applied to the MDPS reduction gear module—the driving power source to assist steering rotation—which adds a constant extra load. Thus, structural stability to compensate for the additional load is required, which is why structural analysis was carried out for the designed worm wheel. Figure 3 shows the 3D model, as well as details of the helical-type of worm wheel structure for structural analysis. The CAE analysis model for the spur and the worm wheel were created in the same method. The structural analysis S/W applied to this study is ABAQUS 6.10. The load condition in the structural analysis was set to 60 N m at the room temperature condition, the maximum torque that may be generated when applied to compact and family sedan vehicles (engine displacement: ~1600–2000 cc). The contact condition between the worm wheel and gear was set to “surface-to-surface”. The tooth form part (Figure 3a), middle locking part (Figure 3b) and end part (Figure 3c) were set to “tie (bonded)” condition. Figure 3d,e shows the CAE analysis model with boundary conditions and finite element model with “tetra-hedra mesh type”. The stress and strain that the gear tooth profile and the glass fiber reinforcement content (weight fraction) added to the polyamide (PA66) material for polymeric worm wheel production were subject to analysis. Table 2 shows the material properties of glass fiber-reinforced polyamide according to glass fiber contents used in this study. A polyamide is highly hygroscopic and the mechanical properties vary according to the moisture absorption. Generally, since the molded products or components with glass fiber-reinforced polyamide are used in various humid environments, the mechanical properties in the moisture condition are considered for securing of product specification. Table 3 shows the stress and strain analysis results of the designed gear tooth profile and frame. As the same amount of torque was applied, the worm and worm wheel tended to make contact at two points, and as the same worm wheel material was used, the contact area of the helical-type of worm wheel was larger. As the contact area was small, the load was concentrated at a certain point, which could be disadvantageous in terms of durability. As to the maximum stress and strain at the tooth profile part, the helical-type indicated 100 MPa, 0.028, and the spur type 120 MPa, 0.03, which shows that the value of the spur type is relatively high. It was the same in the case of the frame part, which indicates that the helical-type of worm wheel is advantageous in terms of worm wheel durability. As to stress and strain analysis results in consideration of the glass fiber reinforcement content, the helical worm wheel was chosen as the subject and the result is shown in Table 4. The maximum stress and strain with a large amount of glass fiber reinforcement content present were relatively small for both the gear tooth profile and the frame parts, and the difference was very small. It is judged, therefore, that as glass fiber reinforcement content increases, the brittleness and strength of the molded part increase accordingly.

**Table 2:** Material properties of the glass fiber-reinforced polyamide according to fiber contents.

*Item*	*UNIT*	*PA66 + GF25 wt %*	*PA66 + GF50 wt %*
***# Mechanical properties (Cond.)***			
Tensile creep modulus (1 h)	MPa	5,000	10,000
Tensile creep modulus (10 h)	MPa	4,100	8,000
Charpy impact strength (+23 °C)	kJ/m^2^	90	110
Charpy impact strength (–30 °C)	kJ/m^2^	47	90
Charpy-notched impact strength (+23 °C)	kJ/m^2^	12	20
Charpy-notched impact strength (–30 °C)	kJ/m^2^	11	14
***# Thermal properties (Dry)***			
Melting temperature (10 °C/min)	°C	263	262
Glass transition temperature (10 °C/min)	°C	80	80
Temp. of deflection under load (0.45 MPa)	°C	260	262
Temp. of deflection under load (1.80 MPa)	°C	245	260
Vicat softening temperature (50 °C/h 50N)	°C	255	255

**Table 3:** Strain-stress comparison analysis of spur *vs.* helical tooth form worm wheel.

*Item*	*Spur Tooth Form Worm Wheel (PA66 + GF25 wt %)*	*Helical Tooth Form Worm Wheel (PA66 + GF25 wt %)*
Tooth Form Part	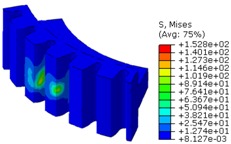	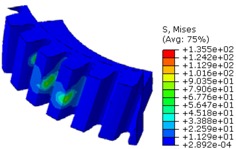
Maximum Stress, Strain	120 MPa, 0.03	100 MPa, 0.028
Middle Locking Part (Frame)	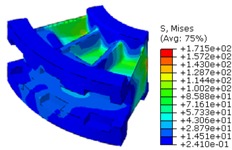	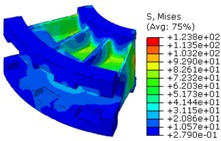
Maximum Stress, Strain	118 MPa, 0.029	98 MPa, 0.027
Tooth Form Part	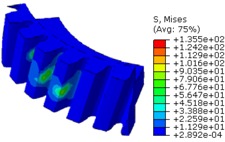	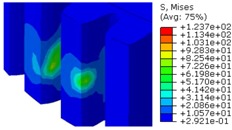
Maximum Stress, Strain	100 MPa, 0.028	86 MPa, 0.023

**Table 4:** Strain-stress comparison analysis according to glass fiber contents.

*Item*	*Helical Tooth Form Worm Wheel (PA66 + GF25 wt %)*	*Helical Tooth Form Worm Wheel (PA66 + GF50 wt %)*
Middle Locking Part (Frame)	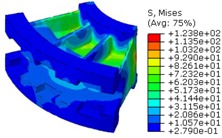	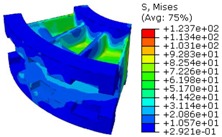
Maximum Stress, Strain	98 MPa, 0.027	93 MPa, 0.026
End Part (Frame)	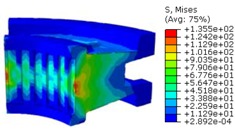	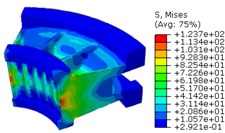
Maximum Stress, Strain	126 MPa, 0.031	119 MPa, 0.029

### 2.2. Design of Injection Mold for Plastic Worm Wheel

**Figure 4:**
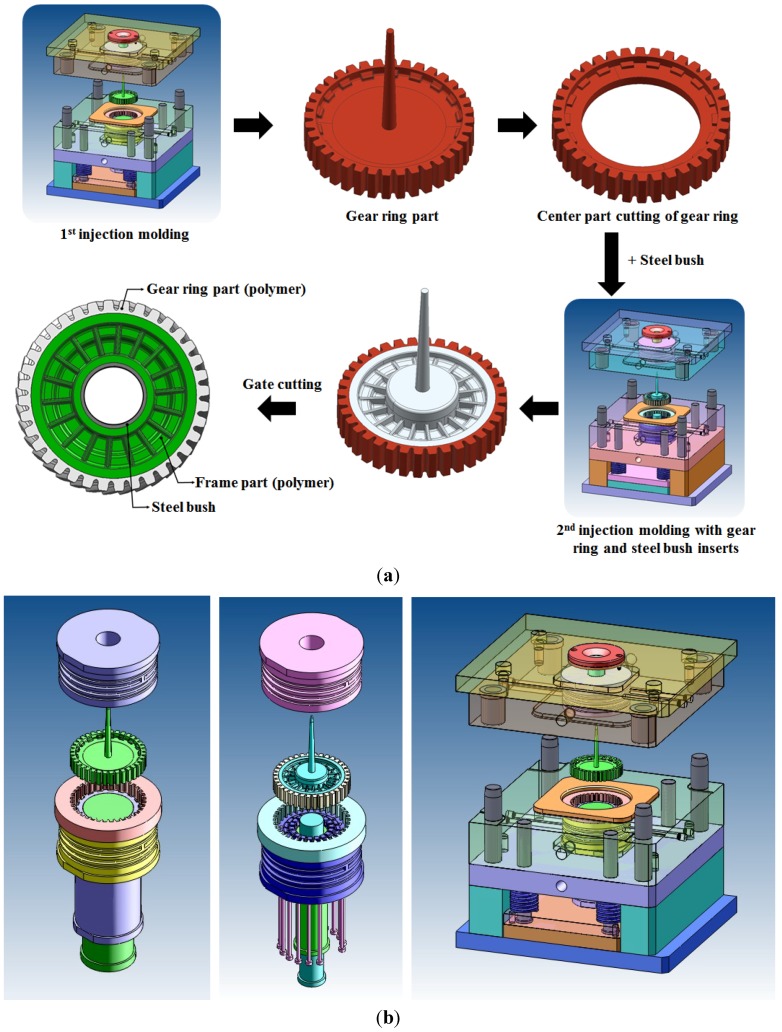
Injection mold components design for plastic worm wheel fabrication (**a**) design of injection molding process for plastic worm wheel; (**b**) design of insert core and mold structure.

The designed plastic worm wheel involves two steps of injection molding, as in Figure 4a. In the first injection molding, the tooth profile part—the gear ring—is molded; the central area of the gearing is removed through the cutting process, and then the worm wheel frame is molded through the second injection molding in which the metal bush combined with the rotation axis and the gear ring produced in the first injection are applied to the insert. This is the general process of making a plastic worm wheel in this study. Figure 4b shows the core and molding plan of the first and second injection molding steps in producing a plastic worm wheel. As the materials applied to the plastic worm wheel in this study include glass fiber reinforcement to secure sufficient strength, the orientation characteristic of the glass fiber reinforcement in reflection of the resin flow are shown. In addition, as the weld mark is likely to weaken the local hardness, the gate location needs to be carefully selected in consideration of the molded part’s function. As for the designed plastic worm wheel, the gate may be put at the center of the wheel or the sides where the tooth profile is located. As the gate is located at the side of the product or the molded part, it may be advantageous regarding the number of cavities, which is related to productivity, but disadvantageous at the same time in that the weld mark is formed at the gear tooth profile part and the glass fiber reinforcement orientation properties become disadvantageous to the worm wheel driving. Thus, this study adopts the top-gate type, as in Figure 4, to secure fiber orientation properties for the load applied to the gear tooth profile. In addition, this prevents the weld line from being formed at the gear tooth profile part.

**Figure 5:**
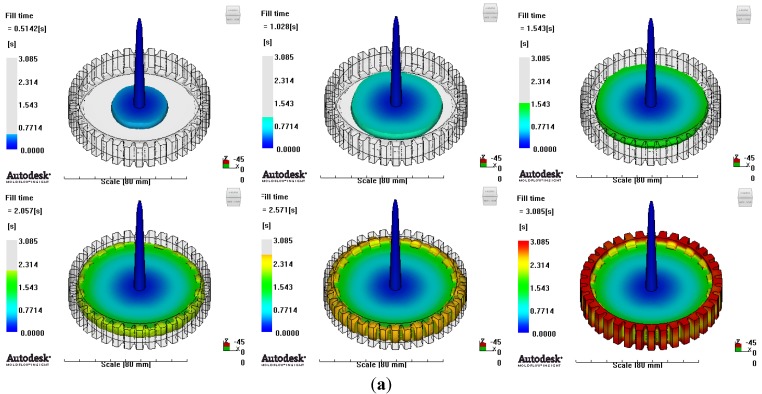

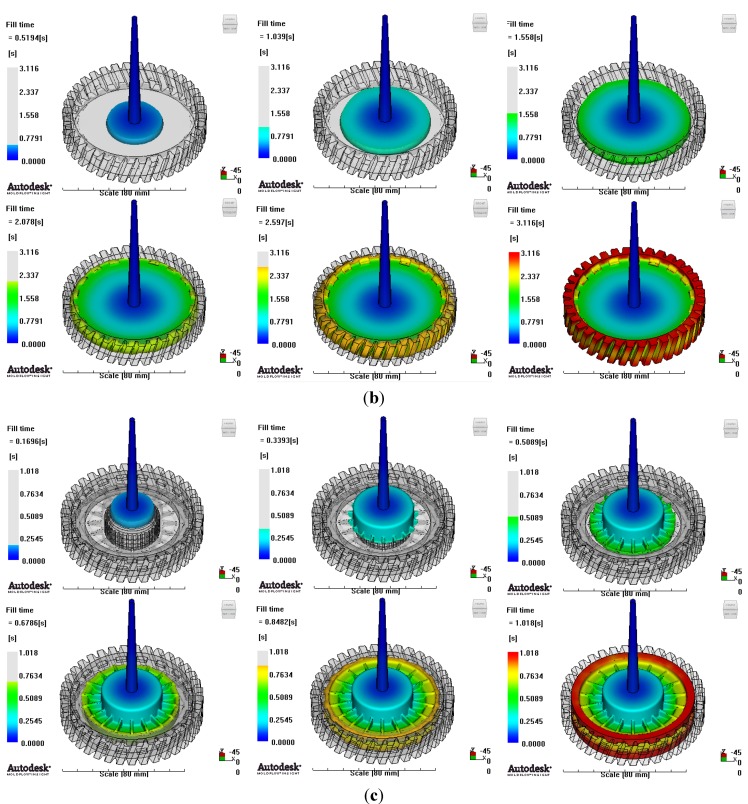
Resin-filling pattern analysis of plastic worm wheel. (**a**) Injection-filling pattern of spur tooth form worm wheel; (**b**) Injection-filling pattern of helical tooth form worm wheel; (**c**) Injection-filling pattern of worm wheel frame part.

In reference to the structural analysis, implemented earlier, with the glass fiber reinforcement content applied, PA66 resin with a glass fiber reinforcement content of either 25 wt % or 50 wt % was applied to the gear ring in the injection molding CAE analysis, and the simulation results for each of the contents of the glass fiber reinforcement were comparatively analyzed. As to the frame part, PA66 resin with a glass fiber reinforcement content of 50 wt % was applied to secure high structural strength, and then the deformation after molding was analyzed. The molding condition applied to the injection molding CAE analysis is shown in Table 5. Figure 5 shows the result of the resin flow analysis that was part of the injection molding CAE analysis carried out on the design. The flow analysis result indicates that both the gear ring and frame parts showed excellent filling tendencies regardless of the type of resin, and that the spur and helical gear rings had the same filling tendencies.

**Table 5:** Strain-stress comparison analysis according to glass fiber contents.

*Item*	*UNIT*	*PA66 + GF25 wt % (Gear ring)*	*PA66 + GF50 wt % (Gear ring, Frame)*
Injection time	s	3	1.5
Packing time	s	20	40
Packing pressure	%	80% of Max. injection pressure	80% of Max. injection pressure
Melt temperature	°C	290	290
Mold temperature	°C	110	110

**Figure 6:**
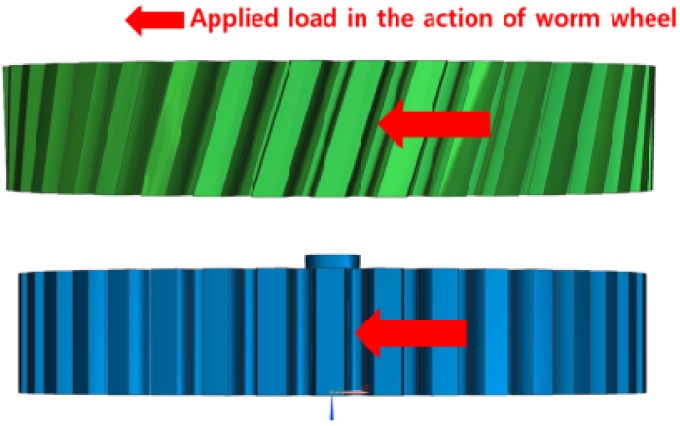
Load direction and position in the action of worm wheel.

Generally, the fiber orientation in the resin fluid core layer cannot be controlled by mold structure because the fiber orientation in the core layer changes in the cooling process without pressure. However, the fiber orientation in the skin layer is decided by the consolidation of resin and does not change in the cooling process. In the case when the feature of the gear ring and injection mold structure designed in this study are considered, the principal direction orientation in the part of tooth, that is, the resin flow direction, has a major effect on the stiffness of the plastic worm gear tooth form. The reason is that as many glass fibers to force applied to the pitch surface is large (Figure 6).

**Figure 7:**
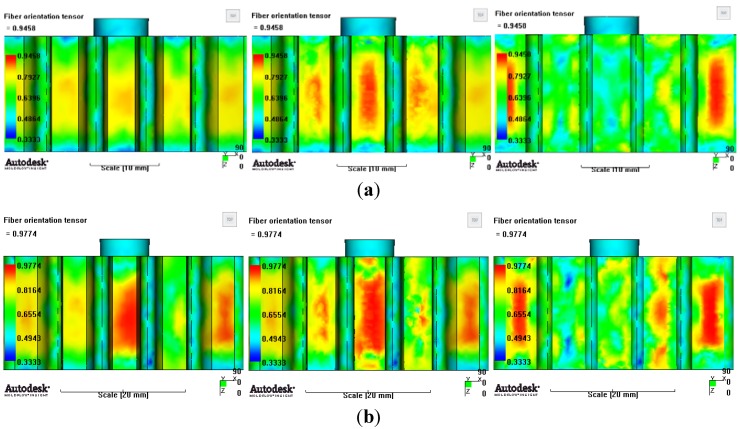
Glass fiber orientation analysis of spur worm wheel: (**a**) Upper, center and low layer from the top of tooth (PA66 + GF25 wt %); (**b**) Upper, center and low layer from the top of tooth (PA66 + GF50 wt %).

**Figure 8:**
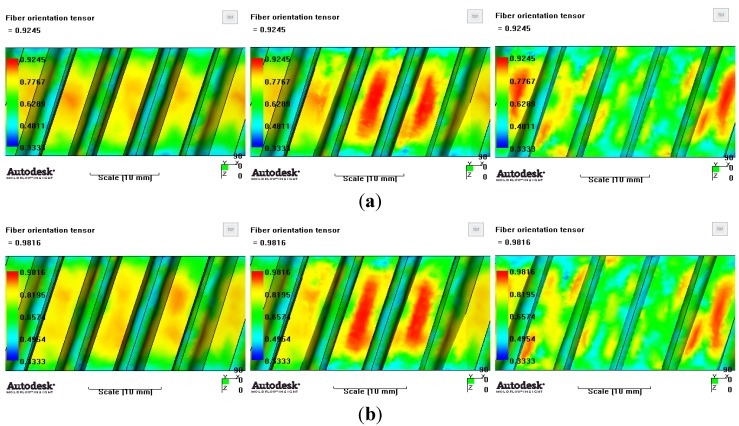
Glass fiber orientation analysis of helical worm wheel: (**a**) Upper, center and low layer from the top of tooth (PA66 + GF25 wt %); (**b**) Upper, center and low layer from the top of tooth (PA66 + GF50 wt %).

Figure 7 and Figure 8 show the prediction results of glass fiber principal direction orientation according to the contents of glass fiber reinforcement. If the red color shaded part in the skin layer is large in the analysis result of fiber orientation, it means that the many glass fibers are oriented to the principal direction and the area of loaded. In reference to the fiber orientation analysis, the fiber principal direction orientation of tooth part in the skin layer is large in the PA66 resin with glass fiber reinforcement content of 25% because the resin fluid in the case of glass fiber reinforcement content of 50% cannot make relatively good progress. However, all cases have poor distribution of glass fiber in the part of the dedendum circle. This result means that the tooth root part of worm wheel may be vulnerable to the force applied to worm wheel teeth compared with the pitch surface part. As to the deformation analysis in connection with the measurement accuracy of the final molded part, the spur and helical gear rings showed the same level of deformation (Table 6).

**Table 6:** Deformation values of *X*-, *Y*-, and *Z*-directions.

Item	*X*-direction	*Y*-direction	*Z*-direction
PA66 + GF25 wt % Spur-type gear ring	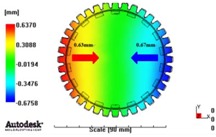	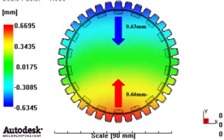	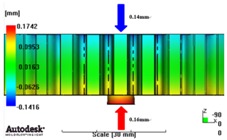
	Max. 0.67 mm	Max. 0.66 mm	Max. 0.16 mm
PA66 + GF25 wt % Helical-type gear ring	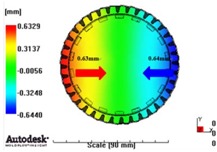	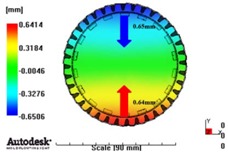	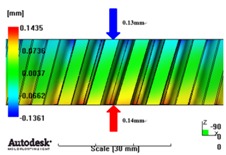
	Max. 0.64mm	Max. 0.65mm	Max. 0.14mm
PA66 + GF50 wt % Spur-type gear ring	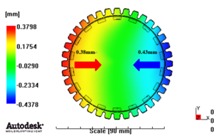	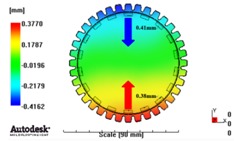	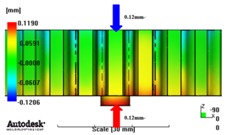
	Max. 0.43mm	Max. 0.41mm	Max. 0.12mm
PA66 + GF50 wt % Helical-type gear ring	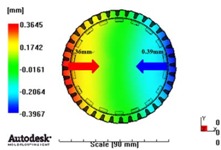	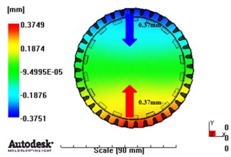	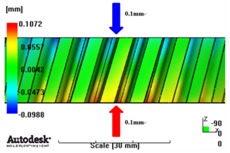
	Max. 0.49mm	Max. 0.37mm	Max. 0.1mm

## 3. Injection Molding Process and Evaluation of Plastic Worm Wheel

**Figure 9:**
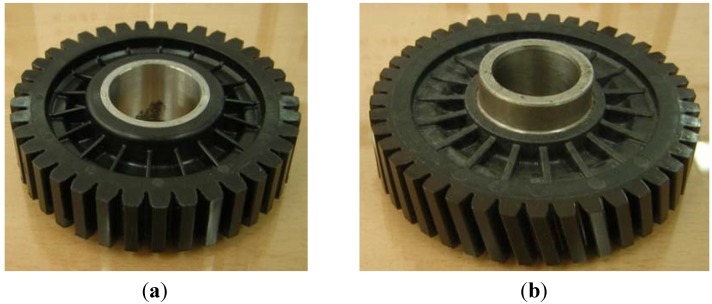
Plastic worm wheels manufactured by injection molding. (**a**) Plastic worm wheel with spur tooth form; (**b**) Plastic worm wheels manufactured by injection molding.

**Figure 10:**
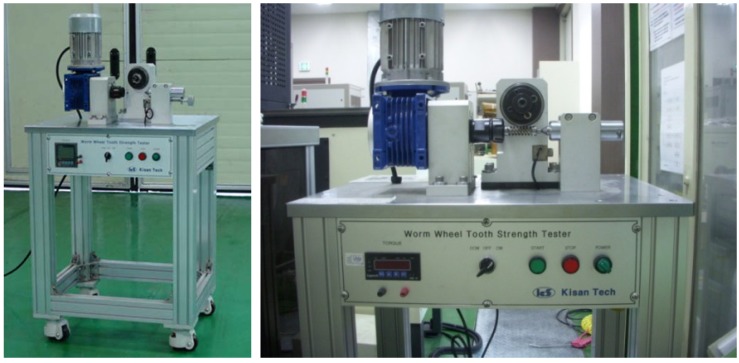
Teeth strength of worm wheel test system.

Table 7 shows the injection molding conditions to produce the sample of spur and helical-type worm wheels. The injection mold conditions were determined in reference to the injection molding CAE analysis. In the case of the gear ring, the conditions were the same as those of the structural and injection molding CAE analysis: the glass fiber-reinforced content was 25 wt % (Minlon 73GM40, DuPont Inc., Ulsan, Republic of Korea) and 50 wt % (Durethan AKV50H2.0, LANXESS), and 50 wt % in the case of injection mold for the frame part with the gear ring as the insert. Figure 9 shows images of the spur and helical worm wheels produced through injection molding as samples. Table 8 shows the material properties of plastic resin used in plastic worm wheel teeth part and teeth strength measured by the exclusive test system. In the case of the teeth strength test, the load-cell data measured when the tooth of worm wheel is broken after assembling the worm gear and worm wheel in the test system. Average strength of teeth part is about 900 kgf/cm^2^, measured using the teeth strength test system (Figure 10).

**Table 7:** Detailed injection molding conditions for plastic worm wheel.

*Item*	*UNIT*	*PA66 + GF25 wt %*	*PA66 + GF50 wt %*
Injection machine capacity	ton	300	300
Cylinder diameter	Ø	55	55
Maximum injection pressure	kgf/cm^2^	724	724
Temperature	°C	270–290	270–290
Injection pressure	kgf/cm^2^	140	140
Injection time	s	3	1.5
Packing time	s	20	40
Mold temperature	°C	110	110
Holding pressure	kgf/cm^2^	140	140
Cooling time	s	120	120

**Table 8:** Material properties of plastic worm wheel prototype (teeth part, PA66 + GF25 wt %).

*Item*	*UNIT*	*Prototype 1*	*Prototype 2*
Hardness(external part)	HRM	101	100
Hardness(internal part)	HRM	97	98
Specific gravity	–	1.32	1.32
Young’s modulus	×10^9^ N/m^2^	85	85
Coefficient of expansion	μm/°C	0.42	0.42
Teeth’s strength	kgf/cm^2^	926	865

**Figure 11:**
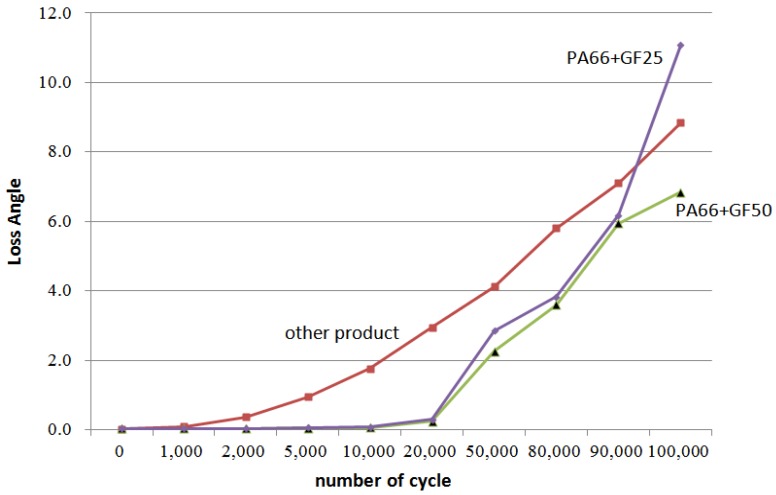
Variation of plastic worm wheel loss angle according to cycle.

As the worm wheel delivers power generated by the motor in order to assist with steering, this might cause the car steering function to be lost upon excessive backlash or damage. Thus, it is vital to conduct a durability evaluation for the applied load. As shown in Table 9, therefore, a system to measure the backlash that might take place during worm wheel operation was developed, and durability with 100,000 repetitions of the same operation was evaluated. The durability evaluation was conducted for the helical-type worm wheels that were judged advantageous in terms of maximum stress and strain after the structural analysis described in the previous section. The existing common worm wheels of high-viscosity resin and the sample worm wheels that contained 25 wt % or 50 wt % glass fiber reinforcement content in this study were comparatively analyzed in terms of backlash occurrence. Figure 11 shows a graph of backlash extents according to the number of repetitions of the operation. While existing worm wheels of high-viscosity resin tended to show a drastic increase of wheel backlash after the 1000th operation, the worm wheel with glass fiber reinforcement showed an increase of wheel backlash after the 20,000th operation. In the case of the worm wheel of 25 wt % glass fiber reinforcement content, however, the wheel backlash increased a great deal more than the worm wheel of high-viscosity resin after the 90,000th operation. It is judged that this resulted from the crack at the gear tooth profile, as in Table 10.

**Table 9:** Durability test conditions of worm wheel.

*Item*	*UNIT*	*VALUE*	*Photo of test system*
Temperature	°C	23	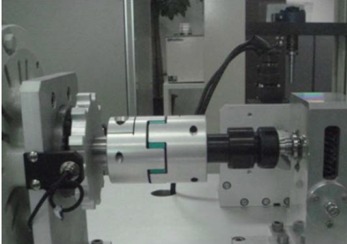
Humidity	%	55	
Velocity	cpm	6	
Load	Nm	60	
Rotation degree	degree	±540	
Cycle(No-pause)	cycle	100,000	
Temperature	°C	23	
Number of article	ea	2	

**Table 10:** Surface images of plastic worm wheel tooth part according to cycle.

*Item*	*PA66 + GF25 wt %*	*PA66 + GF50 wt %*
Internal organization	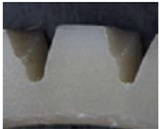	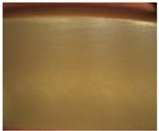
Glass Fiber Feature **(Diameter 6–8 μm, Length 120–160 μm)**	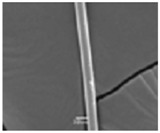	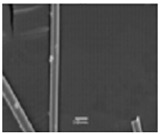
Surface after 5000 cycle	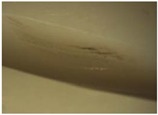	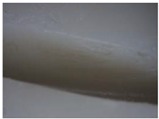
Surface after 30,000 cycle	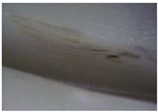	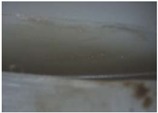
Surface after 50,000 cycle	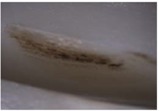	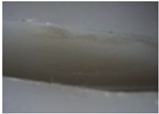
Fracture face	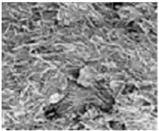	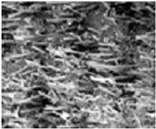

**Figure 12:**
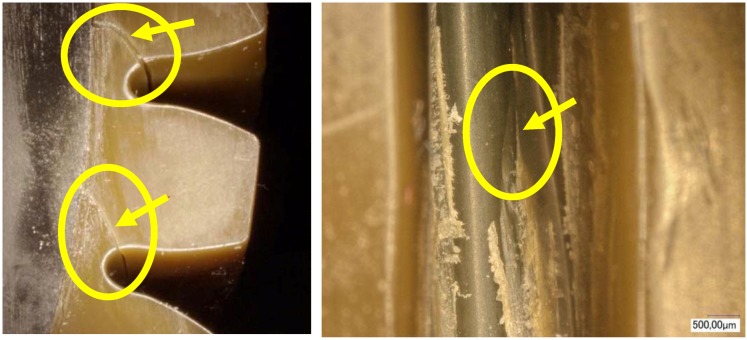
Fracture and crack generation in the durability test.

The difference in wheel backlash according to glass fiber reinforcement content is shown in the surface images of the gear tooth profile in Figure 12. As in the graph in Figure 11, the operation of the worm wheel of 25 wt % glass fiber reinforcement content caused wheel backlash to a relatively large degree after the 50,000th operation, which is also indicated in Figure 9 and shows the extent of the surface crack at the gear tooth profile after the 50,000th operation. Thus, in the durability evaluation, it turned out that worm wheels with glass fiber reinforcement are advantageous compared to those of high-viscosity resin, and that worm wheels of 50 wt % glass fiber reinforcement content are the most advantageous in terms of durability.

## 4. Summary and Discussion

This study develops and evaluates the durability of the plastic worm wheel of a MDPS reduction gear applicable to compact and family sedan vehicles. In the case of plastic worm wheels, the strength and hardness are comparatively inferior to those of metal materials, while they are advantageous in terms of light weight, vibration and noise reduction, and corrosion resistance. Thus, plastic materials with glass fiber reinforcement added were applied to worm wheel production, which was followed by structural analysis and injection molding CAE analysis. As to the structural analysis, it turned out that the helical-type worm wheel is relatively advantageous in terms of the maximum stress and strain in application of the plastic worm wheel tooth profile and the adopted materials. Based on the result of structural analysis, worm wheel materials that were advantageous in terms of structure were chosen, and injection molding CAE analysis was implemented. As a result, it turned out that the worm wheel of 50 wt % glass fiber reinforcement content was advantageous in terms of deformation, which has a great effect on the operation of the worm wheel. After the CAE analysis, an injection mold was produced that was, in turn, used to produce a plastic worm wheel. Finally, a durability evaluation on the worm wheel backlash in relation to glass fiber reinforcement content and in comparison with existing high-viscosity resin worm wheels was implemented. that the findings demonstrated that the helical worm wheel of 50 wt % glass fiber reinforcement content is relatively advantageous in terms of performance.

Based on the CAE analysis and the results of the tests conducted in this study, it was shown that as glass fiber reinforcement content increases, strength and hardness potentially improve, but damage may result from the shock due to fluctuating external force as the brittleness also increases. In addition, as the distribution of glass fiber reinforcement is not even, the strength and hardness of the worm wheel tooth profile might change, which results in quality deterioration. Therefore, future study needs to address injection mold and molding techniques that can secure stable fabric distribution and orientation in addition to methods that ease the determination of the amount of glass fiber reinforcement best suited for the performance of the worm wheel.
